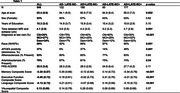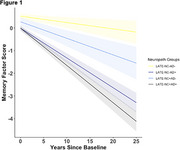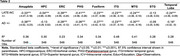# Multicohort characterization of brain volume and cognitive decline in LATE‐NC cases with or without Alzheimer's disease pathology

**DOI:** 10.1002/alz70862_109749

**Published:** 2025-12-23

**Authors:** Leslie S. Gaynor, Alaina Durant, Shubhabrata Mukherjee, Michael L. Lee, Seo‐Eun Choi, Phoebe Scollard, Michael L Cuccaro, David A. A. Bennett, Lisa L. Barnes, Julie A Schneider, Walter W. Kukull, Sarah Biber, Shannon L Risacher, Christos Davatzikos, Guray Erus, Yuhan Cui, Mohamad Habes, Gary W Beecham, Thomas J. Montine, Derek B. Archer, Logan Dumitrescu, Paul K Crane, Renaud La Joie, Angela L. Jefferson, Timothy J. Hohman

**Affiliations:** ^1^ Vanderbilt University Medical Center, Nashville, TN USA; ^2^ University of Washington, Seattle, WA USA; ^3^ Université de Bordeaux, INSERM, Bordeaux France; ^4^ University of Miami Miller School of Medicine, Miami, FL USA; ^5^ Rush University Medical Center, Chicago, IL USA; ^6^ Rush Alzheimer's Disease Center, Rush University Medical Center, Chicago, IL USA; ^7^ National Alzheimer's Coordinating Center, University of Washington, Seattle, WA USA; ^8^ Indiana University School of Medicine, Indianapolis, IN USA; ^9^ University of Pennsylvania, Philadelphia, PA USA; ^10^ University of Texas Health San Antonio, San Antonio, TX USA; ^11^ Wake Forest University School of Medicine, Winston‐Salem, NC USA; ^12^ Stanford University School of Medicine, Stanford, CA USA; ^13^ University of California, San Francisco, San Francisco, CA USA

## Abstract

**Background:**

Limbic‐predominant age‐related TDP‐43 encephalopathy neuropathologic change (LATE‐NC) is a research priority given its high prevalence in dementia cases with and without Alzheimer’s disease pathology (AD). In the absence of LATE‐NC biomarkers, characterizing the clinical presentation of LATE‐NC would improve our ability to identify LATE‐NC cases during life. The present study evaluated the relationship of pathologically confirmed LATE‐NC with and without AD and medial temporal lobe (MTL) volume and longitudinal cognitive change.

**Method:**

Participants from the AD Sequencing Project Phenotype Harmonization Consortium age 70+ at death (*n* = 846) were examined using harmonized, longitudinal cognitive domain scores (memory, executive function, and language), neuropathology, and genetic data (Table 1). Post‐mortem neuropathology scores (AD+=intermediate to high AD likelihood; LATE‐NC+=TDP‐43 stage 1+) were used to categorize participants. Ante‐mortem 3T brain scans were segmented using a multi‐atlas label fusion method, MUSE. ANOVA and chi‐square analyses compared participant characteristics at the last cognitive visit across groups. Linear regression models assessed the effect of AD and LATE‐NC pathology on MTL subregional volume, adjusting for age, sex, education, time between last visit and autopsy, intracranial volume, and cohort. Linear mixed effects models assessed the effect of group on cognitive decline, additionally adjusting for age*time and race/ethnicity.

**Result:**

Last memory, executive function, and language scores differed by post‐mortem pathology; AD‐LATE‐NC+ and AD‐LATE‐NC‐ groups differed only in memory scores. Longitudinally, all other groups, including AD‐LATE‐NC+, had a faster rate of decline in memory scores on average compared to the AD‐LATE‐NC‐ group (Figure 1; time x AD‐LATE‐NC+=‐0.20, CI [‐0.35, ‐0.06], *p* = 0.01). On average, only AD+ groups had a faster rate of decline in executive function and language scores compared to the AD‐LATE‐NC‐ group. All MTL subregional volumes were associated with both AD and LATE‐NC, except the superior temporal gyrus, which was associated with neither, and the hippocampus, which was associated with LATE‐NC but not AD pathology (Table 3).

**Conclusion:**

In a large, multicohort study, AD and LATE‐NC uniquely were associated with MTL volume and cognitive decline. Characterizing the clinical correlates of LATE‐NC is essential to identifying LATE‐NC during life and supporting future research into its pathogenesis.